# Complex pleural empyema can be safely treated with vacuum-assisted closure

**DOI:** 10.1186/1749-8090-6-130

**Published:** 2011-10-06

**Authors:** Zsolt Sziklavari, Christian Grosser, Reiner Neu, Rudolf Schemm, Ariane Kortner, Tamas Szöke, Hans-Stefan Hofmann

**Affiliations:** 1 Department of Thoracic Surgery, Hospital Barmherzige Brüder Regensburg, Prüfeningerstraße 86, 93049 Regensburg, Germany; 2 Department of Thoracic Surgery, University Regensburg, Franz-Josef-Strauss-Allee 11, 93053 Regensburg, Germany

## Abstract

**Objective:**

For patients with postoperative pleural empyema, open window thoracostomy (OWT) is often necessary to prevent sepsis. Vacuum-assisted closure (VAC) is a well-known therapeutic option in wound treatment. The efficacy and safety of intrathoracal VAC therapy, especially in patients with pleural empyema with bronchial stump insufficiency or remain lung, has not yet been investigated.

**Methods:**

Between October 2009 and July 2010, eight consecutive patients (mean age of 66.1 years) with multimorbidity received an OWT with VAC for the treatment of postoperative or recurrent pleural empyema. Two of them had a bronchial stump insufficiency (BPF).

**Results:**

VAC therapy ensured local control of the empyema and control of sepsis. The continuous suction up to 125 mm Hg cleaned the wound and thoracic cavity and supported the rapid healing. Additionally, installation of a stable vacuum was possible in the two patients with BPF. The smaller bronchus stump fistula closed spontaneously due to the VAC therapy, but the larger remained open.

The direct contact of the VAC sponge did not create any air leak or bleeding from the lung or the mediastinal structures. The VAC therapy allowed a better re-expansion of remaining lung.

One patient died in the late postoperative period (day 47 p.o.) of multiorgan failure. In three cases, VAC therapy was continued in an outpatient service, and in four patients, the OWT was treated with conventional wound care. After a mean time of three months, the chest wall was closed in five of seven cases. However, two patients rejected the closure of the OWT. After a follow-up at 7.7 months, neither recurrent pleural empyema nor BPF was observed.

**Conclusion:**

VAC therapy was effective and safe in the treatment of complicated pleural empyema. The presence of smaller bronchial stump fistula and of residual lung tissue are not a contraindication for VAC therapy.

## 1. Introduction

Thoracic empyema, the inflammatory process in a preformed anatomical space, defined by the visceral and parietal pleura, was one of the first recognised thoracic pathological entities that had therapeutic challenge: "Ubi pus, ibi evacua". As a paradoxical result of increased life expectancy, improved survival of malignant diseases and extended operability criteria within and outside the scope of thoracic surgery, the pool of potential candidates for pleural empyema is expanding [1]. In addition, antibiotic abuse has led to increased numbers of therapy-resistant cases. Despite significant advances in the treatment of thoracic infections, empyemas remain a problem in modern thoracic surgery. The overall mortality after postoperative pleural empyema can reach 26% [2].

For many patients, especially with postpneumonectomy empyema or BPF, chest tube insertion or thoracoscopic/open debridement fails to control the infection and ends in sepsis. In these cases, open window thoracostomy (OWT) should be offered [3]. Marsupialisation of the cavity via rib(s) resection and open drainage is a well-established method with low risk [4]. It can be applied either as a definite treatment with intent to cure, a preliminary procedure prior to definite treatment or as a last resort procedure when others have failed to achieve a relatively stable disease state [1]

Since the introduction of vacuum assisted closure therapy (VAC therapy), increasing indications for the treatment of acute or chronic wound infections can be found [5]. Thoracic application, especially in patients with poststernotomy infections, is also well accepted [6]. The first reports of intrapleural VAC therapy were published in 2006 [7]

We have reviewed our experience concerning the management of pleural empyema with VAC therapy after performing an OWT. In particular, the question of VAC application in patients with BPF or remaining lung tissue was of specific interest.

## 2. Patients and Methods

### 2.1. Study sample

In this retrospective study we investigated eight patients with multimorbidity (Karnofsky index < 50%), treated for a postoperative or recurrent pleural empyema between October 2009 and July 2010. We excluded patients who received VAC therapy for mediastinitis after cardiac surgery or for chest wall abscesses not involving the pleural space. The Ethics Commission at the Krankenhaus der Barmherzigen Brüder Regensburg approved the study.

### 2.2 Patient demographics

Of 414 operated patients, six patients developed postoperative empyema (incidence: 1.5%) between October 2009 and July 2010. One patient had a recurrent postpneumonic empyema, the remaining patient was referred from an outside institution.

All patients were men with a mean age of 66.1 years and a range of 53 to 76 years. Patient demographics and lung pathologies are summarised in Table 1. Four patients had lung cancer and two of them received induction chemotherapy, specifically radio-chemotherapy. The resection of the tumour included one pneumonectomy, two lobectomies and one lower bilobectomy. After primary resection, the pathologist demonstrated three R0 and one R1 resection. The patient with R1 resection received subsequent restpneumonectomy because of BPF.

**Table 1:** Demographics of patients

Variable	P1	P2	P3	P4	P5	P6	P7	P8
Age	66♂	71♂	67♂	76♂	74♂	69♂	53♂	53♂

Karnofsky Index < 50%	Yes	Yes	Yes	Yes	Yes	Yes	Yes	Yes

Diagnosis	NSCLC Stage II a	Chronic rib fracture	NSCLC Stage y III a	Atelectasis	Postpneumonic empyema	Emphysema	NSCLC Stage III a	NSCLC Stage y II b

Neoadjuvant Therapy	No	No	Radiochemo.	No	No	No	No	Chemo.

Primary Operation	Lobectomy R0	Chest wall Stabilisation	Lobectomy R0	Decort.	Decort. (thoracoscopic)	Volume Reduction	Bilobectomy R1	Pneumectomy R0

Pathophys. of Empyema	Postop.	Postop.	Postop.	Postop.	Recurrent	Postop.	Postop.	Postop.

Onset	Acute	Chronic	Acute	Chronic	Chronic	Acute	Acute	Acute

Bronchopleural Fistula	Yes	No	No	No	No	No	Yes	No

Number of Interventions before OWT and VAC	2	1	1	0	0	1	1	0

Art of Intervention	Restpneum. Débridement	Débridement	Chest Tube	-	-	Chest Tube	Restpneu.	-

Microbiological Infection	Strep. Staph.	Staph.	Staph.	Staph. Pseudo.	Strep.	Enterobac. Asperg.	Staph. Asperg.	Staph.

P: Patient, NSCLC: Non-small cell lung cancer, Decort.: Decortication, BPF: Bronchopleural Fistula, Multimorbid.: Multimorbidity, Strep.: Streptococcus, Staph.: Staphylococcus, Asperg.: Aspergillosis, Acute Empyema: < 30 days, Chronic Empyema > 30 days., Restpneum.: Restpneumectomy, Pathophys.:Pathophysiology

The other postoperative empyemas resulted after one chest wall reconstruction with rib resection (fracture) and one lung volume reduction (emphysema). Two decortications were performed (one atelectasis, one empyema).

Five patients presented an early/acute (≤ 30 days after primary thoracotomy, with a mean of 24.7 days) and three patients a late/chronic pleural empyema (> 30 days, with a mean of 68 days). Only two patients (25%) had detectable BPF due to bronchial stump dehiscence. In five of eight patients, an initial intervention for treatment of the detected empyema was performed (Table 1.). Independent from the time of empyema, *Staphylococcus, Streptococcus*, and anaerobic species were the most frequently isolated organisms. Additionally, *Aspergillus fumigatus *was found in two patients.

### 2.3 Surgical procedure (OWT and VAC therapy)

**Figure 1:**
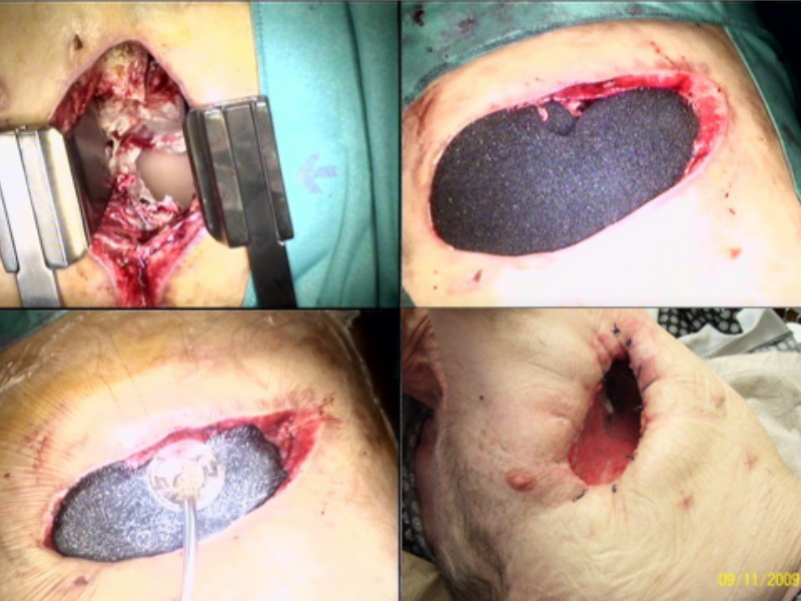
**Intrathoracic vacuum closure**.

The operation for OWT and VAC included the resection of 2-4 ribs, pus evacuation, debridement, flushing the cavity with ringer solution and 10% Betaisodona (Povidon-Iod, Mundipharma) solution (Figure 1.). Suturing the skin flaps on the margins of the OWT constituted the thoracostoma. The VAC sponges (black GranuFoam Standard Dressings, 400 - 600 microns) were inserted in the residual pleural cavity through the thoracostoma (Figure 1.) to fill the entire pleural space. The sponges covered the leakage directly; no membranes were used for the BPF or the remaining lung.

For the procedure, we worked with a vacuum system from KCI Medical (Wiesbaden, Germany). Suction was set to -100 mmHg from the start (maximum suction -125 mmHg), but in two patients with pneumonectomy, the initial suction was -75 mmHg. The sponges were changed once or twice a week, depending on the incorporation of the granulation tissue into the sponges. Only a small amount of debridement was required at each sponge change.

## 3. Results

### 3.1 Time of OWT and VAC

The indication for OWT and VAC intervention was acute sepsis, failed primary surgical intervention (e.g., tube insertion) or complications of primary interventions. The mean time between primary thoracotomy and OWT was 52 days (range 21 days to 126 days).

In five patients, either chest tube drainage or rethoracotomy with restpneumectomy/debridement initiated the empyema treatment (Table 2.). Four patients underwent one initial intervention before the fenestration and vacuum closure, and one patient had two interventions. In two patients, a detectable BPF was dissected, directly closed by stitches and covered by a pericardial flap during the first intervention. All five patients received the OWT and VAC secondarily because of failed initial empyema treatment. Direct creation of OWT with VAC therapy was performed in three patients.

**Table 2:** VAC and outcomes

Variable	P1	P2	P3	P4	P5	P6	P7	P8
Immediate/delayed Creation of OWT	Delayed	Delayed	Delayed	Immediate	Immediate	Delayed	Delayed	Immediate

Number of Interventions before OWT and VAC	2	1	1	0	0	1	1	0

Art of Intervention	Restpneum. Débridement	Débridement	Chest Tube	-	-	Chest Tube	Restpneu.	-

Indication of OWT+VAC	Sepsis	Bleeding Fistula	Failed primary Th.	Osteomyelitis	Fistula	Failed primary Th.	Sepsis	Muscle necrosis

P.o. mechanical ventilation after VAC	Yes	No	No	No	No	Yes	Yes	No

Number of VAC Changes in OR	4	2	2	1	0	5	3	0

Max. Suction mm Hg	- 75	- 125	- 125	- 125	- 100	- 100	- 75	- 125

Hospitalization in days after VAC	22	45	17	15	14	38	47 (exitus)	8

Antibiotic Therapy, in days	10	12	7	6	7	19	47	6

Clinical outpatient VAC	No	No	No	Yes	Yes	No	-	Yes

Outcome	Healed	Healed	Healed	Healed	Healed	Healed	Died of Sepsis	Healed

Closing planned	Yes	Yes	Yes	Yes	Yes	Yes	-	Yes

Chest wall closed	No*	No*	Yes	Yes	Yes	Yes	-	Yes

OWT Duration, in days	not closed	not closed	51	39	31	164	-	59

P: Patient number, P.o.: postoperative, Max.: maximal, OR.: Operation room, *: closing was planned, but patient rejected it.

The mean time between first intervention and OWT with VAC therapy was 18.4 days for directly treated patients and 33.5 days for patients with delayed OWT with VAC therapy.

### 3.2 Course of VAC therapy

**Figure 2:**
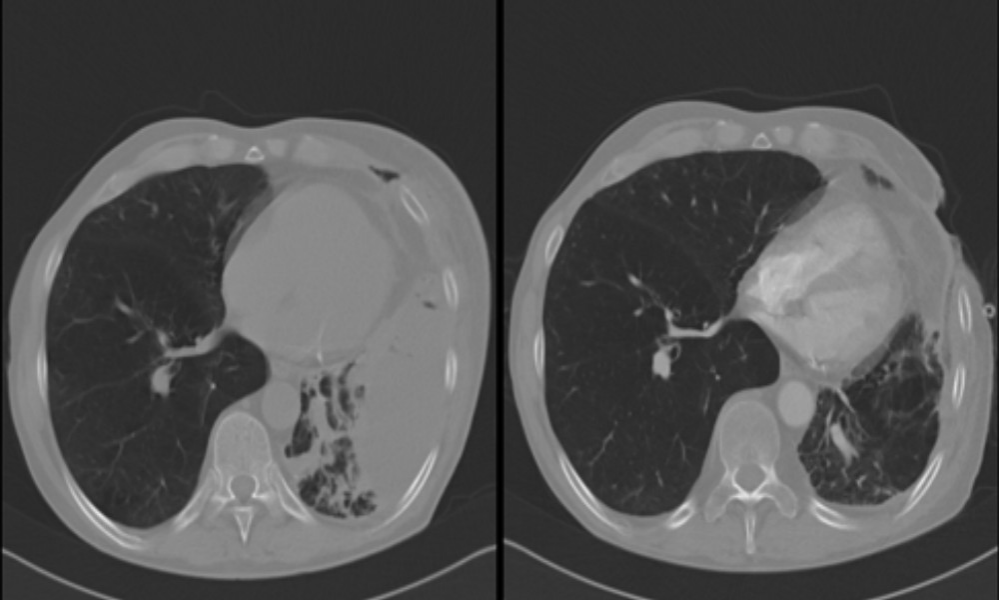
**Radiologic demonstration; VAC dressing could help expand dystelectatic lung**.

Local control of the infection and control of sepsis was satisfactory in seven of the eight patients treated by OWT and VAC therapy. The patients tolerated a suction of 75-125 mm Hg and did not reacted with arrhythmia or haemodynamic complications due to the traction on the mediastinum during attempts to increase the suction. Membranes for the protection of the lung parenchyma were not necessary. Furthermore, the suction used did not create any air leak or bleeding from the lung or the mediastinal structures. At the time of OWT and VAC installation, three patients were in severe clinical conditions with acute respiratory insufficiency with mechanical ventilation. One patient was resucitated. After implementing VAC therapy, two patients could be weaned from ventillatory support after one and five days. In patients with residual lung tissue, VAC therapy allowed improved re-expansion of the residual lung. This expansion could be well radiologic demonstrated. (Figure 2.)

In both patients with detectable BPFs, these fistulas remained following the first intervention. At this time, the recurrent BPFs were one millimetre and eight millimetres, and closing was not possible in either case. However, both patients with BPF underwent successful local treatment of pleural empyema with sufficient suction. The smaller bronchus stump fistula closed spontaneous from VAC therapy, but the larger remained open.

In the beginning of the VAC therapy, dressing changes were performed under anaesthesia in the operating theatre, with a mean rate of 2.1 changes and a range of 0 to 5 changes. Additional changes were set individually and performed without analgesic two or three times a week. Antibiotic therapy was stopped when the microbiological culture did not show any further pathogenic bacteria colonisation (mean antibiotic therapy: 16.3 days).

### 3.3 Outcome of VAC-therapy

Seven of the eight patients (87.7%) were successfully treated by OWT and VAC therapy. One patient died in the late postoperative period (day 47 p.o.) of fulminant aspergillum sepsis-related multiorgan failure. Although he was the patient with the persistent eight millimetres BPF, the thoracic cavity of this patient was sterile during VAC treatment and his death was due to other factors.

The success of VAC therapy was defined by discharging the patients in good health with a Karnofsky Index of 70% and with a non-infected pleural cavity. In most cases the dimension of the pleural cavity was also decreased by OWT and VAC therapy. The mean hospital stay after OWT and VAC installation was 22.7 days. Four patients left our hospital without VAC, and the cavity was filled with dry dressing material. Three patients were transferred with VAC to the outpatient service. Despite ambulant VAC therapy, these patients had a good quality of life and excellent mobility.

In all patients, the closing of the OWT was planned, and after a mean time of three months (97.5 ± 66.5 days), the chest wall was closed in five patients. The surgical closure was performed after obliteration of the pleural cavity with muscle transposition (M. pectoralis N = 2, M. serratus anterior N = 1). In two patients, the secondary closure was performed without thoracoplasty because of maximal contraction of the pleural cavity. Two patients subsequently rejected the closure of the OWT, the last follow-up (after 15 respectively 18 months) did not show sign of recurrent infection.

After follow-up at an average of 7.7 months (range of 4 to 12 months), neither pleural empyema nor BPF recurred in any of the seven surviving patients. All of these patients reported a very good quality of life in an outpatient interview.

## 4. Discussion

The often-cited Latin aphorism *"Ubi pus, ibi evacua" *suggests that clinicians should open infected cavities. We showed that the combination of traditional OWT with the new intrathoracic VAC therapy fulfilled the criteria of this old knowledge, especially in debilitated patients with complicated empyema.

In regards to VAC therapy for open wound management, this new technique is often discussed as a reserve treatment when there are no other options. In one VAC group reported by Palmen and colleagues [8], the OWT was delayed 58 ± 119 days after the diagnosis of the empyema. Once treatment commenced, the total duration of OWT with VAC therapy was 31 ± 19 days. In the present study, for comparison, patients with delayed OWT and VAC therapy left our hospital after 31 ± 14 days and one patient died. In patients with initial fenestration, however, the hospital stay was only 11.5 ± 3.5 days. This finding was consistent with Massera and colleagues [9], who concluded that immediate creation of OWT is a significant predictor of successful thoracostomy closure. We subscribed to this opinion and extended early OWT installation to combined VAC therapy. In our opinion, the alternative treatment of OWT and VAC therapy should be discussed as soon as possible, especially for postoperative or chronic pleural empyema and in patients with increased risk for impaired wound healing (e.g., diabetes, obesity, steroids).

The presence of BPF or remaining lung tissue is not a contraindication for VAC therapy. Groetzner and colleagues [10], as well as Palmen and colleagues [8], defined patients with BPF as not qualified for VAC therapy. This recommendation led to Aru and colleagues [11]. closing all of the BPFs before application of the VAC system. The closure of a BPF is the best precondition of empyema treatment, but sometimes the second closure is not possible. We treated two BPF patients with VAC and in all the installation of vacuum was possible. In one patient with a one mm fistula, the BPF was sufficiently closed after VAC therapy. The other BPF, with a diameter of eight millimetres, could not be closed by VAC, which was not a problem in the VAC treatment. Future studies should investigate the diameter of BPF that can be closed by negative pressure in VAC therapy.

VAC therapy seems to have a beneficial effect on the re-expansion of the remaining lung in patients (Figure 2.). For example, two patients with respiratory insufficiency were quickly removed from their respirators after VAC therapy.

Similar to other reports [5,8,10,11], we applied a maximum suction of -125 mmHg directly to the pulmonary tissue using the V.A.C. GranuFoams. Starting with a lower suction (-75 mmHg) was useful in patients with prior pneumonectomy. In addition, membranes for tissue protection were not necessary and no major complications related to vacuum-assisted management were observed.

The frequency and the location of intrathoracic VAC varies, as this part of the surgical treatment is not defined. For example, Palmen and colleagues [8]. changed the system in the surgical ward without anaesthesia every 3^rd ^to 5^th ^day, or more depending on purulent secretion or increased infection. However, Aru and colleagues [11]. performed all sponge changes under general anaesthesia. For comparison, our patients underwent two debridements and VAC changes in the operation room, and additional changes were performed every 3^rd ^to 5^th ^day in the ward.

In most cases, VAC therapy resulted in the rapid eradication of local infection. We therefore withdrew antibiotics when there were no signs of sepsis and the thoracic cavity became sterile (mean time of 16.3 days). However, the role of simultaneous antibiotics flushing (e.g., V.A.C. Instill) has not yet been investigated.

After treatment of sepsis and local control of the empyema, often with reduction of the pleural cavity, patients could be discharged to an outpatient service with initial daily wound care by specialized nurse technicians. It was occasionally useful to continue the VAC therapy in this ambulant sector with the aim of further reduction of the pleural cavity (in the present study, N = 3). Thoracic surgeons should perform this outpatient treatment weekly.

In follow-up visits, the indication for closure of the OWT should be periodically evaluated. We closed our OWT after a mean time of three months, but two patients rejected this procedure. For comparison, Matzi and colleagues [12]. performed closure of the thoracic cavity after VAC therapy in all cases between the 9th and 48th day (mean of 22 days). Additionally, Groetzner and colleagues [10]. used the VAC system as a bridge to reconstructive surgery and removed it after a mean period of 64 +/- 45 days (range of 7 to 134 days) in all patients. These patients underwent direct surgical wound closure, and complete healing without recurrence was achieved in 11/13 (85%) patients.

Data from the literature show that the interval between installation and closure of the OWT is considerable longer in patients without additional VAC therapy [8,13]. The average duration of OWT without VAC therapy at the Maastricht University Medical Centre was 933 ± 1422 days [8]. Maruyama and colleagues reported an OWT interval from 128 +/- 32, 1 to 365, 8 +/- 201 days, depending on indication [13]. In our patients with VAC therapy the chest wall was closed after a mean time of three months (97.5 ± 66.5 days). In the non-VAC group of Palmen and colleagues [8]. six of the eight patients could be discharged home. In only two of them the OWT was closed by muscular flap. Four patients died during follow-up because of OWT-related complications (massive bleeding n = 1, recurrent infections of the thoracic cavity n = 3).

The rate of successful empyema treatment and closure of OWT by reconstructive surgery is in our study as well as in other studies with VAC therapy [10,12]. substantial higher in correlation to groups with only OWT treatment.

In our opinion, the closure of the OWT depends on the patient's individual situation (e.g., general condition of the patient, planned rehabilitation). As a final step, the closure of the chest guarantees full mobilisation and a good quality of life, with only a very low risk of recurrent infections.

### 4.1. Study Limitations

We were only able to recruit eight patients who had required an OWT and only five patients who had residual pulmonary parenchyma in the past year. Because of these small numbers of patients, this study is a series of case studies and not a randomised trial.

## 5. Conclusion

Patients with complicated empyema were successfully treated with OWT and VAC therapy, so the use of this procedure should be discussed early. The most important advantages of the OWT with VAC were fast treatment of sepsis and local control of the pleural cavity. Suction therapy could also improve pulmonary function (re-expansion). In addition, the presence of bronchial stump fistulas or residual lung tissue is not a contraindication for vacuum-assisted closure. Furthermore, the length of hospitalization was shorter in patients with immediate OWT and VAC-therapy installation, and outpatient treatment with VAC-therapy is possible.

## Competing interests

The authors declare that they have no competing interests.

## Authors' contributions

CG, RS, RN and AK participated in the design of the study. TS participated in the sequence alignment and drafted the manuscript. ZS and HH conceived of the study and participated in its design and coordination. All authors read and approved the final manuscript.

## References

[B1] Molnar TF Current surgical treatment of thoracic empyema in adults Eur J Cardiothorac Surg 2007 32 422 30 10.1016/j.ejcts.2007.05.028 17646107

[B2] Lemmer JH Botham MJ Orringer MB Modern management of adult thoracic empyema J Thorac Cardiovasc Surg 1985 90 849 55 4068734

[B3] Light RW A new classification of parapneumonic effusions and empyema Chest 1995 108 299 301 10.1378/chest.108.2.299 7634854

[B4] Deslauriers J Jacques LF Gregoire J Role of Eloesser flap and thoracoplasty in the third millennium Chest Surg Clin N Am 2002 12 605 23 10.1016/S1052-3359(02)00017-0 12469491

[B5] Renner C Reschke S Richter W Thoracic empyema after pneumonectomy: intrathoracic application of vacuum-assisted closure therapy Ann Thorac Surg 2010 89 603 4 10.1016/j.athoracsur.2009.06.037 20103353

[B6] Sjogren J Malmsjo M Gustafsson R Ingemansson R Poststernotomy mediastinitis: a review of conventional surgical treatments, vacuum-assisted closure therapy and presentation of the Lund University Hospital mediastinitis algorithm Eur J Cardiothorac Surg 2006 30 898 905 10.1016/j.ejcts.2006.09.020 17056269

[B7] Varker KA Ng T Management of empyema cavity with the vacuum-assisted closure device Ann Thorac Surg 2006 81 723 5 10.1016/j.athoracsur.2004.10.040 16427885

[B8] Palmen M van Breugel HN Geskes GG van Belle A Swennen JM Drijkoningen AH Open window thoracostomy treatment of empyema is accelerated by vacuum-assisted closure Ann Thorac Surg 2009 88 1131 6 10.1016/j.athoracsur.2009.06.030 19766795

[B9] Massera F Robustellini M Pona CD Rossi G Rizzi A Rocco G Predictors of successful closure of open window thoracostomy for postpneumonectomy empyema Ann Thorac Surg 2006 82 288 92 10.1016/j.athoracsur.2005.11.046 16798231

[B10] Groetzner J Holzer M Stockhausen D Tchashin I Altmayer M Graba M Intrathoracic application of vacuum wound therapy following thoracic surgery Thorac Cardiovasc Surg 2009 57 417 20 10.1055/s-0029-1185907 19795330

[B11] Giorgio M Aru MD Nicholas B Jew Curtis G Tribble MD Walter H Merrill MD Intrathoracic Vacuum-Assisted Management of Persistent and Infected Pleural Spaces Ann Thorac Surg 2010 90 266 71 10.1016/j.athoracsur.2010.04.092 20609790

[B12] Matzi V Lindenmann J Porubsky C Mujkic D Maier A Smolle-Juttner FM V.A.C.-treatment: a new approach to the management of septic complications in thoracic surgery Zentralbl Chir 2006 131 Suppl 1 S139 40 16575665 10.1055/s-2006-921474

[B13] Maruyama Riichiroh Ondo Kaoru Mikami Koji Ueda Hitoshi Motohiro Akira Clinical Course and Management of Patients Undergoing Open Window Thoracostomy for Thoracic Empyema Respiration 2001 68 606 610 10.1159/000050580 11786716

